# Surgeon–Anesthesiologist Relationship in the Operating Room: A Cross-Sectional Survey of Surgeons’ Perceptions

**DOI:** 10.7759/cureus.105264

**Published:** 2026-03-15

**Authors:** Samia Saidi, Safae Dehbi, Anas Elhanbali, Saad Elharrak, Larbi Dafali, Salma Ech Cherif El Kettani, Hicham Ziani, Hamza Talbi, Noussaiba Nabil, Aziza Bentalha, Alae El Koraichi

**Affiliations:** 1 Pediatric Intensive Care Unit, Ibn Sina University Hospital, Rabat, MAR; 2 Department of Pediatric Anesthesiology, Ibn Sina University Hospital, Rabat, MAR

**Keywords:** anesthesiologists, collaboration, operating room teamwork, perioperative communication, surgeon’s experience

## Abstract

Background

Effective collaboration between surgeons and anesthesiologists is essential for patient safety and efficient operating room workflow. However, communication challenges, organizational constraints, and stress may strain this relationship and affect perioperative care.

Methods

We conducted a cross-sectional survey of surgeons from various specialties using an anonymous structured questionnaire. The survey assessed organizational factors, quality of professional relationships, perceived anesthesiologist stress, communication practices, and overall satisfaction. Data were analyzed descriptively.

Results

Seventy-six surgeons participated, mainly from university hospitals. Missing equipment (75.7%), workload, and anesthesia-related delays were frequently reported as negatively affecting collaboration. Although familiarity with the same anesthesiologists was considered beneficial, 32.8% reported insufficient clarity of patient information and 57.9% questioned preoperative investigations. Most surgeons (81.6%) perceived excessive anesthesiologist stress, which was associated with impaired communication and workflow. Many reported modifying their communication or surgical approach in response.

Conclusion

Organizational constraints, communication, and stress significantly influence surgeon-anesthesiologist collaboration, with potential implications for patient safety and perioperative practice.

## Introduction

Effective collaboration between anesthesiologists and surgeons is a cornerstone of safe, efficient, and high-quality surgical care. These two specialties are inherently interdependent throughout the perioperative period, with each professional’s actions directly influencing patient outcomes. The anesthesiologist is responsible for maintaining physiological stability, managing pain, and addressing comorbidities, while the surgeon is expected to ensure procedural efficiency, respect operative timing, and conduct a predictable surgical course. This relationship has been described in the literature as one of critical interdependence, in which optimal coordination is essential for both clinical performance and organizational efficiency [1].

Despite this interdependence, relational challenges between surgeons and anesthesiologists remain common. Previous studies have highlighted multiple sources of tension within the operating room environment [2]. Differences in professional training and clinical priorities constitute a major contributor: surgeons are primarily focused on the technical execution and outcome of the procedure, whereas anesthesiologists emphasize global patient management, physiological safety, and risk prevention. These distinct perspectives may generate divergent expectations and decision-making approaches. In addition, organizational constraints such as operating room scheduling, surgical delays, emergency cases, and preoperative assessment requirements frequently lead to conflicts related to time management and patient prioritization [3].

Hierarchical dynamics, whether real or perceived, further complicate this relationship, particularly in situations involving intraoperative incidents and responsibility attribution. Communication failures have been consistently identified as a leading cause of adverse events in the operating room, including inadequate anticipation of anesthetic requirements, limited participation in briefings and debriefings, and impaired communication under stress [3,4]. Such breakdowns not only affect team cohesion but may also compromise patient safety.

In this context, a better understanding of the surgeon-anesthesiologist relationship is essential. While previous research has largely examined this collaboration from a multidisciplinary or anesthesiologist-centered perspective, fewer studies have specifically explored surgeons’ perceptions. Therefore, this study aimed to evaluate surgeons’ perceptions of their professional relationship with anesthesiologists in the operating room, with particular attention to organizational constraints, communication dynamics, and perceived stress, and to explore how these factors may influence surgical workflow and patient safety outcomes.

## Materials and methods

Study design and setting

A cross-sectional descriptive survey was conducted among surgeons from multiple surgical specialties to assess their perceptions of the professional relationship with anesthesiologists in the operating room. The study was conducted between October 2025 and December 2025. Data were collected using an anonymous questionnaire distributed in both electronic and paper-based formats. The study was reported in accordance with the Strengthening the Reporting of Observational Studies in Epidemiology (STROBE) guidelines for cross-sectional studies.

Participants

Eligible participants included practicing surgeons of both sexes and from various surgical specialties. Participation was voluntary and open to surgeons working in university teaching hospitals, regional hospitals, and the private sector. A total of 76 surgeons participated in the study. The majority were practicing in university teaching hospitals, with additional respondents from regional hospitals and the private sector. No personal or identifiable information was collected in order to ensure anonymity. Participants were recruited using a convenience sampling approach, based on voluntary participation among surgeons reachable through professional networks and hospital departments during the study period.

Data collection instrument

Data were collected using a structured questionnaire comprising 22 items divided into five thematic sections: (i) Human and material resources in the operating room; (ii) Quality of the professional relationship with anesthesiologists; (iii) Surgeons’ perceptions of anesthesiologists’ stress and its impact on surgical activity; (iv) Overall satisfaction with interprofessional collaboration; (v) Transparency and completeness of information communicated during the perioperative period.

The questionnaire was developed following a review of the literature addressing operating room teamwork, interprofessional collaboration, and surgeon-anesthesiologist relationships. The initial items were designed to explore organizational, relational, and psychological aspects of collaboration in the operating room.

To assess content relevance and clarity, the preliminary questionnaire was reviewed by a panel of clinicians including surgeons and anesthesiologists. The reviewers evaluated the clarity, comprehensibility, and relevance of each item, and minor modifications were made based on their feedback. This expert review process aimed to ensure the content validity of the instrument prior to distribution.

The final questionnaire consisted of 22 items grouped into five thematic sections.

It consisted primarily of single-choice and multiple-choice items. An open-ended comment section was included to allow participants to provide additional qualitative insights.

Data collection procedure

The electronic version of the questionnaire was disseminated primarily through professional messaging groups using the WhatsApp® platform, with a link directing participants to an online survey form. Additional responses were collected using printed questionnaires distributed in person within hospital departments. Participation was voluntary, responses were anonymous, and no incentives were provided.

Ethical considerations

Participation in the study was voluntary, and informed consent was implied by completion of the questionnaire. No personal or identifiable information was collected. The study was conducted in accordance with the ethical principles outlined in the Declaration of Helsinki.

Statistical analysis

Data were analyzed using Jamovi statistical software (version 2.6.24). Categorical variables were summarized as frequencies and percentages, and continuous variables were expressed as means ± standard deviations. Given the exploratory and descriptive nature of the study, no inferential statistical analyses or hypothesis testing were performed, and therefore, p-values and test statistics were not calculated. The study aimed to provide a descriptive overview of surgeons’ perceptions rather than to test specific associations.

Sample size justification

The sample size was determined pragmatically based on surgeon availability during the study period. Given the descriptive and exploratory objectives of the study, a formal sample size calculation was not performed. A total of 76 respondents provided an initial exploratory overview of surgeons’ perceptions and allowed the identification of recurring themes and potential areas of concern regarding surgeon-anesthesiologist collaboration. Similar survey-based studies in perioperative and interprofessional research have reported comparable sample sizes for exploratory analyses.

## Results

General characteristics of the study population

A total of 76 surgeons participated in the survey. The mean age of respondents was 28.9 ± 3.1. Male surgeons represented 55 of 76 participants (72.4%), while 21 of 76 (27.6%) were female. Regarding professional experience, 67 of 76 respondents (88.2%) had less than five years of practice, whereas 9 of 76 (11.8%) had five or more years of experience. Seventy participants practiced in university teaching hospitals (92.1%), while four (5.3%) worked in regional hospitals and two (2.6%) practiced in the private sector (Table 1).

**Table 1:** Demographic and professional characteristics of the study population (N = 76) Data are presented as number (percentage). No inferential statistical tests were performed.

Characteristic	Value (N = 76)
Sex^a^	
Male	55 (72.4)
Female	21 (27.6)
Professional experience^a^	
< 5 years	67 (88.2)
≥ 5 years	9 (11.8)
Type of practice setting^a^	
University teaching hospital	70 (92.1)
Regional hospital	4 (5.3)
Private sector	2 (2.6)

Human and material resources

Several organizational factors were reported as potentially affecting surgeons’ well-being and professional interactions. Missing or inadequate equipment was reported by 58 of 76 respondents (75.7%). Heavy workload or multiple weekly shifts were reported by 18 of 76 surgeons (24.3%). Lack of punctuality of anesthesiologists at the start of procedures was reported by 15 of 76 respondents (20.1%). Anesthesia-related delays were perceived as negatively affecting interpersonal relationships with anesthesiologists by 58 of 76 participants (78.4%) (Table 2).

**Table 2:** Surgeons’ perceptions of organizational factors, communication, and anesthesiologist stress (N = 76) Data are presented as number (percentage). No inferential statistical tests were performed.

Domain	Item	Value (N =76)
Organizational and material factors	Missing or inadequate equipment	58 (75.7)
	Heavy workload/multiple shifts per week	18 (24.3)
	Lack of punctuality of anesthesiologists	15 (20.1)
	Anesthesia-related delays negatively impacting interpersonal relationship	58 (78.4)
Relationship and communication	Regularly work with the same anesthesiologists	54 (71.1)
	Familiarity improves surgical workflow	64 (84.2)
	Insufficient clarity or honesty regarding patient information	25 (32.8)
	Doubts about relevance of preoperative paraclinical investigations	44 (57.9)
Perceived anesthesiologist stress	Perceived work overload or stress	62 (81.6)
	Stress considered excessive	38 (50.0)
	Negative impact on workflow fluidity	30 (39.5)
	Negative impact on communication	28 (36.8)
	Negative impact on surgeon’s performance	9 (11.8)
	Modification of surgical approach due to anesthesiologist stress	46 (60.5)
	Do not feel fully free to share all patient data	47 (61.8)
	Considered omitting or minimizing patient information	40 (52.6)
	To avoid surgery cancellation or reporting	31 (40.5)
	Belief omission would not affect patient safety	26 (35.1)
	To save time	18 (24.3)
	Adapted communication to avoid stressing anesthesiologist	58 (77.0)
	Prefer a calm anesthesiologist who communicates during intraoperative incidents	66 (86.7)

Relationship and communication with anesthesiologists

Regular collaboration with the same anesthesiologists was reported by 54 of 76 surgeons (71.1%). Familiarity with the same professionals was perceived as improving surgical workflow by 64 of 76 respondents (84.2%).

However, 25 of 76 surgeons (32.8%) reported insufficient clarity or honesty regarding patient-related information. In addition, 44 of 76 participants (57.9%) expressed doubts, to varying degrees, about the relevance of preoperative paraclinical investigations requested by anesthesiologists. A total of 47 of 76 surgeons (61.8%) reported not feeling completely free to share all patient-related information. Furthermore, 40 of 76 respondents (52.6%) acknowledged having considered omitting or minimizing certain patient data.

Among these, 31 of 76 (40.5%) reported that this was to avoid surgery cancellation or reporting, 18 of 76 (24.3%) to save time, and 26 of 76 (35.1%) believed that such omission would not affect patient safety. Additionally, 58 of 76 surgeons (77.0%) reported adapting their communication style during surgery to avoid increasing anesthesiologist stress. During intraoperative incidents, 66 of 76 respondents (86.7%) expressed a preference for a calm anesthesiologist who actively communicates with the surgical team.

Surgeons’ perception of anesthesiologists’ stress

Perceived work-related stress or overload among anesthesiologists was reported by 62 of 76 respondents (81.6%). Stress was considered excessive by 38 of 76 surgeons (50.0%). Perceived anesthesiologist stress was described as negatively affecting workflow fluidity by 30 of 76 respondents (39.5%), communication by 28 of 76 (36.8%), and surgeons’ own performance by 9 of 76 (11.8%). As a consequence, 46 of 76 surgeons (60.5%) reported modifying their surgical approach during procedures in response to perceived anesthesiologist stress.

## Discussion

This survey provides descriptive insights into surgeons’ perceptions of their professional relationship with anesthesiologists in the operating room. The findings suggest that several organizational and interpersonal factors may influence collaboration between these professionals. In particular, respondents frequently reported issues related to equipment availability, workload, and anesthesia-related delays. Missing or inadequate equipment was the most commonly reported challenge, affecting more than three-quarters of respondents. These findings are consistent with previous literature indicating that resource constraints and excessive workload can contribute to fatigue, frustration, and tension within surgical teams [5]. Such conditions may affect professional interactions and communication between surgeons and anesthesiologists. Qualitative studies have similarly reported that limited resources and organizational stressors are associated with surgeon burnout and strained workplace relationships [6]. In addition, anesthesia-related delays were identified by many participants as a factor negatively affecting collaboration, which aligns with prior research highlighting scheduling and workflow disruptions as potential sources of conflict within operating room teams [7].

Another important observation from this survey concerns the perceived importance of familiarity and continuity between surgeons and anesthesiologists. A large proportion of participants reported regularly working with the same anesthesiologists and believed that this continuity contributed to smoother surgical workflows. Previous studies have shown that stable operating room teams may promote trust, improve coordination, and enhance overall teamwork efficiency [8,9]. However, despite this perceived benefit, a substantial proportion of surgeons reported concerns regarding the clarity or transparency of patient-related information. Such perceptions suggest that communication challenges may still occur even within teams that frequently work together. Earlier research has similarly highlighted that incomplete or unclear information exchange between surgical team members may affect team coordination and performance [10].

In addition, more than half of the respondents expressed doubts regarding the relevance of some preoperative paraclinical investigations requested by anesthesiologists. This observation may reflect differences in professional perspectives and clinical priorities between the two specialties. Surgeons often focus primarily on procedural efficiency and surgical outcomes, whereas anesthesiologists may prioritize comprehensive risk assessment and perioperative safety. When these perspectives are not sufficiently discussed or aligned, misunderstandings or frustration may arise. Previous studies have reported similar tensions related to differing clinical priorities and decision-making approaches within operating room teams [11].

Communication-related challenges also emerged as a prominent theme in this study. A considerable proportion of respondents reported not feeling fully comfortable sharing all patient-related information, and some acknowledged having considered minimizing or omitting certain details. The main reasons reported included concerns about potential surgery cancellation, time constraints, or the perception that the omitted information would not significantly affect patient safety. Although these perceptions may represent pragmatic coping strategies in a high-pressure environment, they raise important concerns regarding transparency and communication practices in the operating room. Previous research has demonstrated that incomplete information transfer is a recognized contributor to adverse events and complications in surgical settings [12-14]. Additionally, many respondents reported adapting their communication style to avoid increasing anesthesiologist stress. While these adjustments may help maintain a calm working atmosphere, they may also create ambiguity or limit open communication. Stress and hierarchical dynamics have previously been shown to impair communication and increase the risk of misunderstandings in complex clinical environments [8,15].

Perceived stress among anesthesiologists was another central theme identified by the respondents. Most surgeons reported observing significant levels of work-related stress among anesthesiologists, which they perceived as potentially affecting workflow and communication within the operating room. According to the respondents, such stress may contribute to reduced communication efficiency, disruptions in workflow, and, in some cases, changes in surgeons’ own intraoperative behavior. These observations illustrate how stress perceived within one professional group may influence the broader team dynamic. Previous studies have similarly reported that high levels of stress among operating room professionals may negatively affect teamwork and increase the likelihood of errors or communication breakdowns [12,13].

During intraoperative incidents, most respondents indicated that they preferred an anesthesiologist who remains calm and communicates clearly with the surgical team. This finding highlights the importance of emotional regulation and effective communication during critical situations. Psychological safety and collaborative crisis management have been widely recognized as essential elements of effective team performance in high-risk clinical environments such as the operating room [16].

Overall, the findings of this study suggest that surgeons perceive organizational constraints, communication practices, and professional stress as important elements influencing surgeon-anesthesiologist collaboration. These observations underscore the potential value of interventions aimed at improving teamwork within the operating room, such as structured communication practices, team training programs, stress management strategies, and organizational improvements related to workload and resource availability. However, given the descriptive nature of the study design, these findings should be interpreted as perceptions reported by surgeons rather than as evidence of causal relationships.

Strengths of the study

This study contributes to the literature on operating room teamwork by specifically exploring surgeons’ perceptions of their collaboration with anesthesiologists. This perspective has been relatively less frequently examined compared with anesthesiologist-centered or multidisciplinary studies. The structured questionnaire allowed the exploration of multiple dimensions of collaboration, including organizational factors, communication practices, perceived stress, and professional satisfaction. In addition, the inclusion of surgeons from different practice settings provided a broader overview of these perceptions within the perioperative environment.

Study limitations

Several limitations should be acknowledged when interpreting the findings of this study. First, the relatively small sample size and the predominance of early-career surgeons may limit the generalizability of the results to broader surgical populations. Second, participants were recruited through a convenience sampling approach, which may introduce selection bias. Third, the study reflects only surgeons’ perceptions and does not include the perspectives of anesthesiologists or other members of the operating room team, which may provide a more comprehensive understanding of interprofessional dynamics. In addition, the reliance on self-reported survey data may introduce response bias or recall bias.

Another limitation relates to the questionnaire used in this study. The survey instrument was specifically developed for the purposes of this research and, although its items were informed by relevant literature and reviewed by clinicians to ensure clarity and relevance, it did not undergo a formal psychometric validation process prior to its use. In particular, quantitative measures of content validity such as the item-level content validity index (I-CVI) and scale-level content validity index (S-CVI), as well as formal reliability testing, were not performed. Consequently, the questionnaire should be considered an exploratory tool designed to capture surgeons’ perceptions rather than a fully validated measurement instrument.

Finally, the analysis was limited to descriptive statistics due to the exploratory nature of the study, and no inferential analyses were performed to examine potential associations between variables. Future research involving larger and more diverse samples, incorporating the perspectives of multiple operating room professionals, and employing formally validated instruments may provide more comprehensive and generalizable insights into surgeon-anesthesiologist collaboration in the operating room.

## Conclusions

This study highlights that surgeons perceive the quality of the surgeon-anesthesiologist relationship as being strongly influenced by organizational constraints, communication practices, and stress management within the operating room. While familiarity between professionals is generally viewed as beneficial, persistent challenges related to workload, resource availability, perceived stress, and communication transparency may negatively affect collaboration and intraoperative decision-making. Addressing these factors through improved communication, shared decision-making, stress management strategies, and institutional support may enhance interprofessional relationships, promote professional well-being, and ultimately contribute to improved patient safety and surgical outcomes.

## Appendices

Bilingual questionnaire (French/English)

French Version

A- Informations générales: 1.Age: 2.Sexe: Femme Homme 3. Où est ce que pratiquez vous en tant que chirurgien?* CHU CHP/CHR En libéral 4. Quelle spécialité chirurgicale? 5. Années d' expérience: Moins de 5ans 5-10 ans 11-20ans Plus de 20ans B-  Moyens humains et matériaux: 1. A quelle fréquence etes-vous capables de temporiser vos interactions avec le personnel notamment l' anesthésiste après une journée chargée ou des gardes fréquentes?* Jamais Rarement Souvent Toujours 2. A quelle fréquence l'anesthésiste se présente en retard  pour débuter une intervention chirurgicale?*

Jamais Rarement Souvent Toujours 3. Ce retard influe-t-il négativement sur votre relation avec l' anesthésiste?* Jamais Parfois Souvent 4. A quelle fréquence trouvez-vous qu'il y a un matériel manquant?* Jamais Rarement Souvent Toujours 5. La non disponibilité du matériel a -t - elle un impact négatif sur votre humeur/ relationnel ?* Rarement Souvent Toujours C-  Relation avec l' anesthésiste: 1. Comment évaluez-vous globalement votre relation professionnelle avec les anesthésistes? (1=très mauvaise ; 5=excellente) 1 2 3 4 5

2. A quelle fréquence travaillez-vous avec les mêmes anesthésistes?* Très rarement Occasionnellement Régulièrement Presque toujours 3. Trouvez-vous que cette familiarité permet un meilleur déroulement de la chirurgie?* Oui (meilleur déroulement) Non (rien ne change) Non (au contraire, elle impacte négativement) 4. Selon votre ressenti, trouvez-vous que l' anesthésiste vous informe de manière complète et claire sur l' état du patient avant l' entrée au bloc?* Jamais Rarement Parfois Souvent Toujours 5. Etes-vous toujours convaincues par les examens complémentaires demandées par l' anesthésiste?(ex: radiographie, bilan, ou chirurgie reportée):* Jamais Rarement Parfois Souvent Toujours 6. Lorsque vous n' êtes pas convaincus, en parles-vous à votre anesthésiste?* Oui, j' en parle à l' anesthésiste et nous en discutons calmement. Non, je'en parle pas et fais ce que j' ai à faire pour ne pas annuler le malade. Indifférent

D- Stress perçu chez l' anesthésiste: 1. A quelle fréquence percevez-vous un stress ou une surcharge de travail chez l' anesthésiste au bloc?* Jamais Rarement Parfois Souvent Très souvent 2. Selon vous, ce stress perçu chez l' anesthésiste est-il* Souvent justifié et aide à améliorer le déroulement de la chirurgie Souvent exagéré et constitue un obstacle au processus de la chirugie 3- Ce stress peut il dérégler (en per-opératoire):* La fluidité du travail? La communication entre vous? Votre propre performance? Aucun impact 4. Avez vous déjà modifié votre approche chirurgicale en raison du stress de l' anesthésiste? (ex: raccourcir le geste chirurgical, manquer de vérifier les points de suture...)* Souvent Rarement Jamais E- Satisfaction globale: 1. Sur une échelle de 1 à 5, quel est votre niveau de satisfaction global concernant votre collaboration avec les anesthésistes  en per-opératoire? (1= pas satisfait , 5=très satisfait)* 1 2 3

4 5 2. Quels aspects de collaboration souhaitez-vous améliorer ?* Communication péri-opératoire Prise de décision conjointe Gestion conjointe des incidents en per-opératoire Partage des responsabilités Reconnaissance mutuelle F- Transparence des données communiquées à l' anesthésiste: 1. Si vous ressentez un manque de transparence de la part de  l' anesthésiste, avez-vous déjà eu l' idée d' omettre ou minimiser certaines données du dossier pour faciliter la PEC du patient? Souvent Rarement Jamais 2. Si oui, pour quelles raisons? Eviter une annulation ou un report Gagner du temps Conviction que cela ne mettrait pas en danger le patient 3. Vous est-il arrivé d'adapter votre discours médical pour éviter de stresser ou de heurter l' anesthésiste en per-opératoire (jugeant que le stress de l' anesthésiste pourrait retentir négativement sur le déroulement de la chirurgie) Souvent Occasionnellement Jamais 4. En cas d' incident per-opératoire, quelle est, pour vous, la situation idéale? Anesthésiste calme qui communique et implique le chirurgien dans son approche Anesthésiste calme qui se concentre uniquement sur son travail et ne communique rien

Anesthésiste stressé qui communique et implique le chirurgien dans son approche Anesthésiste stressé qui se concentre uniquement sur son travail et ne communique rien 6. Dans quelle mesure vous sentez-vous libre de transmettre l' ensemble des données médicales, sans crainte de blocage ou de jugement ? (Par exemple: lésion d' un vaisseau en per-opératoire..) (1= pas du tout libre; 5= totalement libre) 1 2 3 4 5 F- Remarques libres Si vous souhaitez ajouter une remaque, une suggestion ou un témoignage libre: Si vous souhaitez recevoir un résumé des résultats globaux, laissez ci dessous un email anonyme ou professionnel Merci pour votre collaboration 

English Version

A - General Information 1. Age: 2. Sex: - Female - Male

3. Where do you practice as a surgeon? - University Hospital (CHU) - Regional/Provincial Hospital (CHP/CHR) - Private practice

4. Surgical specialty:

5. Years of experience: - Less than 5 years - 5-10 years - 11-20 years - More than 20 years

B - Human and Material Resources

1. How often are you able to moderate your interactions with staff, particularly the anesthesiologist, after a long working day or frequent on-call duties? - Never - Rarely - Often - Always

2. How often does the anesthesiologist arrive late to start a surgical procedure? - Never - Rarely - Often - Always

3. Does this delay negatively affect your relationship with the anesthesiologist? - Never - Sometimes - Often

4. How often do you find that some equipment is missing? - Never - Rarely - Often - Always

5. Does the lack of equipment negatively affect your mood or professional relationship? - Rarely

- Often - Always

C - Relationship with the Anesthesiologist

1. How would you evaluate your professional relationship with anesthesiologists overall? (1 = very poor; 5 = excellent) 1 2 3 4 5

2. How often do you work with the same anesthesiologists? - Very rarely - Occasionally - Regularly - Almost always

3. Do you think this familiarity improves the surgical workflow? - Yes (better workflow) - No (no change) - No (negative impact)

4. Do anesthesiologists provide complete and clear information about the patient before entering the operating room? - Never - Rarely - Sometimes - Often

- Always

5. Are you convinced by the additional tests requested by the anesthesiologist (radiology, lab tests, postponement of surgery)? - Never - Rarely - Sometimes - Often - Always

6. When you are not convinced, do you discuss it with the anesthesiologist? - Yes, we discuss it calmly - No, I proceed without discussing to avoid canceling the patient - Indifferent

D - Perceived Stress of the Anesthesiologist

1. How often do you perceive stress or workload overload in the anesthesiologist in the operating room? - Never - Rarely - Sometimes - Often - Very often

2. In your opinion, this stress is: - Often justified and helps improve surgical workflow

- Often exaggerated and an obstacle to surgery

3. Can this stress affect: - Workflow fluidity - Communication between you - Your own performance - No impact

4. Have you ever modified your surgical approach because of anesthesiologist stress? (e.g., shortening the procedure, not fully checking sutures) - Often - Rarely - Never

E - Overall Satisfaction

1. On a scale from 1 to 5, what is your overall satisfaction with collaboration with anesthesiologists during surgery? (1 = not satisfied, 5 = very satisfied)

1 2 3 4 5

2. Which aspects of collaboration would you like to improve? - Perioperative communication - Joint decision making - Joint management of intraoperative incidents

- Shared responsibilities - Mutual recognition

F - Transparency of Information Shared with the Anesthesiologist

1. If you perceive a lack of transparency from the anesthesiologist, have you ever considered omitting or minimizing information in the patient record to facilitate management? - Often - Rarely - Never

2. If yes, for what reasons? - Avoid cancellation or postponement - Save time - Belief it does not endanger the patient

3. Have you ever adapted your medical communication to avoid stressing the anesthesiologist during surgery? - Often - Occasionally - Never

4. In case of an intraoperative incident, what is the ideal situation? - Calm anesthesiologist who communicates and involves the surgeon - Calm anesthesiologist who focuses only on their work - Stressed anesthesiologist who communicates and involves the surgeon - Stressed anesthesiologist who focuses only on their work

5. To what extent do you feel free to share all medical data without fear of judgment or blockage? (1 = not free at all; 5 = completely free) 1 2 3 4 5

G - Additional Comments

If you would like to add a comment, suggestion, or testimony:

If you would like to receive a summary of the results, leave an anonymous or professional email below.

Thank you for your collaboration.
